# Ferroptosis in Liver Diseases: An Overview

**DOI:** 10.3390/ijms21144908

**Published:** 2020-07-11

**Authors:** Martina Maria Capelletti, Hana Manceau, Hervé Puy, Katell Peoc’h

**Affiliations:** 1 Centre de Recherche sur l’Inflammation, INSERM U1149, Université de Paris, Laboratory of Excellence GR-Ex, 75018 Paris, France; m.capelletti@campus.unimib.it (M.M.C.); hana.manceau@aphp.fr (H.M.); herve.puy@aphp.fr (H.P.); 2 Department of Biotechnology and Biosciences, University of Milano-Bicocca, I-20126 Milan, Italy; 3 Laboratoire de Biochimie, Hôpital Beaujon, APHP, 92110 Clichy, France; 4 Centre Français des Porphyries, Hôpital Louis Mourier, APHP, 92701 Colombes, France

**Keywords:** ferroptosis, cell death, liver, iron metabolism

## Abstract

Ferroptosis is an iron-dependent form of cell death characterized by intracellular lipid peroxide accumulation and redox imbalance. Ferroptosis shows specific biological and morphological features when compared to the other cell death patterns. The loss of lipid peroxide repair activity by glutathione peroxidase 4 (GPX4), the presence of redox-active iron and the oxidation of polyunsaturated fatty acid (PUFA)-containing phospholipids are considered as distinct fingerprints of ferroptosis. Several pathways, including amino acid and iron metabolism, ferritinophagy, cell adhesion, p53, Keap1/Nrf2 and phospholipid biosynthesis, can modify susceptibility to ferroptosis. Through the decades, various diseases, including acute kidney injury; cancer; ischemia–reperfusion injury; and cardiovascular, neurodegenerative and hepatic disorders, have been associated with ferroptosis. In this review, we provide a comprehensive analysis of the main biological and biochemical mechanisms of ferroptosis and an overview of chemicals used as inducers and inhibitors. Then, we report the contribution of ferroptosis to the spectrum of liver diseases, acute or chronic. Finally, we discuss the use of ferroptosis as a therapeutic approach against hepatocellular carcinoma, the most common form of primary liver cancer.

## 1. Introduction

Cell death is the concluding event of all essential cellular processes in living cells [1]. Under certain conditions, this lethal process occurs suddenly, making cells incapable of preventing the spreading of cell death; therefore, this event is referred as “accidental/explosive” or necrotic cell death [1]. In contrast, organisms have developed highly controlled cell death modalities (RCD, regulated cell death). The first example of RCD was apoptosis, and then necroptosis, netosis, entosis and autophagy [1]. Ferroptosis (derived from the Greek word “ptosis”, meaning “a fall”, and Ferrum or iron) was first described in 2012 [1,2]. Iron chelators, such as deferoxamine (DFO), prevent the formation of reactive radical species such as the hydroxyl radical, protecting from cell death. Ferroptosis is defined as an iron-dependent, non-apoptotic type of cell death characterized by the increase of reactive oxygen species (ROS) [3].

Moreover, ferroptosis is morphologically distinguishable from other types of cell death. Ferroptosis plays a critical regulatory role in many diseases (e.g., tumorigenesis, ischemia–reperfusion injury, renal failure, nervous system diseases and hematological diseases). Because of the increasing relevance of ferroptosis, several projects have been undertaken focusing on ferroptosis, which rapidly became the new hotspot of research on the treatment and improvement of prognosis of related disorders [3]. Given the trend of an increasing number of publications on ferroptosis, we firmly believe, as authors, in the need of an extensive and detailed review of this topic. Therefore, in the first part of the article, we give a comprehensive description of the different processes involved, while, in the second part, the focus is on the role of ferroptosis in hepatic diseases.

## 2. Discovery of Ferroptosis

In 2003, a study was conducted to identify new molecules with lethal effects on Ras-mutated cells. Among the 23,550, different chemical compounds screened, NSC146109, renamed erastin, had a selectively lethal effect on Ras-expressing cancer cells [4]. In 2008, two other molecules, Ras-selective poisonous small molecules (RSL3-5), were identified, which kill selectively human foreskin fibroblasts (BJeLR) in a non-apoptotic manner [5]. Inhibitors specific for RCDs cannot undo RSL-induced cell death. In contrast, antioxidants (vitamin E) and iron chelators can block and reverse RSL-induced cell death [1]. Therefore, the term “ferroptosis” refers to an iron-dependent, non-apoptotic cell death characterized by lipid peroxidation [3].

## 3. An Overview of Ferroptosis

In vitro ferroptosis inducer (e.g., erastin) treatment leads to a round shape of tumor cells and their detachment from the flask surface. Ferroptosis is characterized by the presence of small mitochondria with condensed mitochondrial densities, reduction or vanishing of mitochondrial cristae and outer mitochondrial membrane (OMM) rupture [1]. In contrast to apoptosis, ferroptotic cells do not show cell shrinkage, chromatin condensation and formation of apoptotic bodies and disintegration of the cytoskeleton [1]. Moreover, no autophagic vacuoles have been found after treatment with ferroptosis inducers. The main morphological and biochemical features of the different types of cell death are summarized in Table 1. The ferroptotic cell appears morphologically and biochemically distinguishable from the other four types of cells Table 1).

## 4. Mechanisms of Ferroptosis

**Figure 1:**
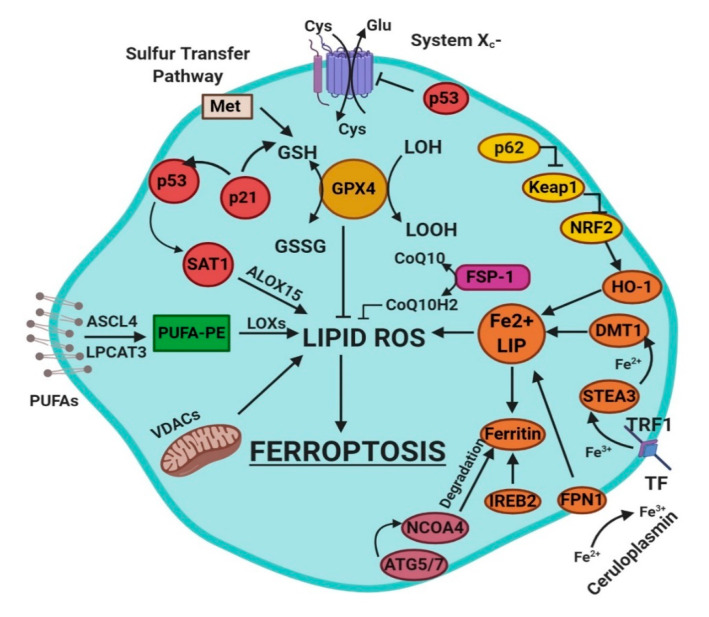
This figure summarizes the regulatory core of ferroptosis, approximately divided into three axes. The first axis is represented in the middle. It includes the GSH/GPX4, sulfur transfer and p53 pathways. The second axis (right part of theF) consists of the iron metabolism pathway, including IREB2 related to ferritin metabolism, the regulation of ATG5-ATG7-NCOA4 pathway and the p62-Keap1-Nrf2 regulatory pathway. These elements can influence the concentration of intracellular iron, mandatory for the development of ferroptosis. On the left, the third axis implies that lipid metabolism p53-spermidine/spermine N1-acetyltransferase 1 (SAT1)-ALOX15, ACSL4, LPCAT3, etc. impact on fatty acids regulation and ferroptosis [3]. Finally, mitochondria are also involved, since VDACs (voltage-dependent anion channels) are inhibited by erastin. In parallel, the independent pathway ferroptosis suppressor protein 1-coenzyme Q10 (FSP-1-CoQ10) acts with GSH/GPX4 to contrast lipid peroxidation [3].

Ferroptosis is a sum of many biological pathways, acting simultaneously. In Figure 1, ferroptosis regulatory pathways are reported. Three main biological axes are roughly:

1. glutathione/glutathione peroxidase 4 (GSH/GPX4) pathway, inhibition of system X_c_^−^, sulfur transfer pathway, and p53 regulatory axis;
2. iron metabolism with the regulation of autophagy protein 5 and 7 (ATG5-ATG7) and nuclear receptor coactivator 4 (NCOA4) pathway and iron-responsive element-binding protein 2 (IREB2) related to ferritin metabolism, and the p62-Kelch-like ECH-associated protein 1 (Keap1)-nuclear factor erythroid 2-related factor (Nrf2) regulatory pathways [3]; and
3. lipid metabolism pathways as p53, arachidonate lipoxygenase 15 (ALOX15), acyl-CoA synthetase long-chain family member 4 (ACSL4), lysophosphatidylcholine acyltransferase 3 (LPCAT3) [3].

### 4.1. Radical Oxygen Species (ROS)

Oxygen (O_2_) is an essential element for aerobic living beings. Cellular membranes are permeable to gas; thus, oxygen permeates freely into the cells, where it undergoes several processes. Oxygen is used in the mitochondria to provide energy through oxidative phosphorylation (OXPHOS) [6]. During cellular respiration and the degradation of organic compounds via graded oxidative reactions, radical oxygen species (ROS) are inevitably produced [7]. Therefore, while essential for life, oxygen participates also in the demise of the organism [8]. Before discussing ROS, it is necessary to define the term “radical”. In chemistry, a radical is an atom, molecule or ion with unpaired highly reactive valence electron. A radical undergoes dimerization reactions spontaneously.

ROS are tightly monitored by cells since they play a pivotal role in tissue homeostasis [9]. In excessive amount, ROS cause cellular toxicity and their concentrations need to be tightly controlled; at the same time, low concentrations of ROS are used as a defensive weapon, since their release in the extracellular environment protects cells from bacterial invasion as well as intracellular signaling molecules. Cellular oxidative stress is defined as a disequilibrium between the oxidative and antioxidant systems, and it appears to be essential in the occurrence of ferroptosis.

Several types of ROS are generated during the ferroptotic process, which can be divided into four main groups: (i) superoxide (O_2_^−^), which is produced because of electron reaction with O_2_ at complex I/III of the mitochondrial electron transport chain and which is rapidly dismutated to hydrogen peroxide (H_2_O_2_) [9]; (ii) hydrogen peroxide (H_2_O_2_), which is moderately reactive and responsible for protein oxidation [9]; (iii) peroxyl radical (OH^•^), which is formed from H_2_O_2_ via the Fenton reaction and is the most reactive ROS [9]; and (iv) lipid peroxides, which are obtained from the oxidation of polyunsaturated fatty acids (PUFAs) [9].

During ferroptosis, the Fenton reaction is the primary source of ROS. The Fenton reaction describes the formation of hydroxide (OH^−^) and hydroxyl (OH^•^) radicals by a reaction between Fe^2+^ and hydrogen peroxide (H_2_O_2_) (Equation (1)).

Fe^2+^ + H_2_O_2_ → Fe^3+^ + OH^−^ + OH (1)

When Henry J.H. Fenton discovered the Fenton reaction, he never mentioned the existence of the hydroxyl radical intermediate (OH^•^). In 1934, Haber and Weiss proposed a modified version of the Fenton reaction. They hypothesized the existence of OH^•^ via reaction of hydrogen peroxide and superoxide with iron as a catalyst. The Haber–Weiss (Equations (2) and (3)) cycle is a two-step reaction, where Fe^3+^ is reduced to Fe^2+^ via reaction with superoxide (O_2_^−^), which in turn reacts with H_2_O_2_ to form OH^−^ and OH^•^, regenerating ferric ion.

O_2_^−^ + Fe^3+^ → Fe^2+^ + O_2_ (2)

Fe^2+^ + H_2_O_2_ → Fe^3+^ + OH^−^ + OH (3)

### 4.2. Regulation of Ferroptosis via Cysteine-Glutathione Redox Axis

Oxidative stress triggers ferroptosis. Redox glutathione (GSH) plays a crucial role in ferroptosis, since it represents the ideal substrate for glutathione peroxidase 4 (GPX4) and is indispensable for preventing ferroptosis. In vitro ferroptosis is typically induced after cysteine starvation, GSH depletion and inhibition of GPX4. Intracellular cysteine concentration is usually maintained low partly due to its cytotoxic effects; regardless, cysteine is directly required for the synthesis of the glutathione tripeptide (Cys-Gly-Glu). Additionally, cysteine is a versatile molecule; cells carry out conversions of the amino acid into a variety of reactive compounds such as taurine, cysteamine, coenzyme A and Fe-S clusters.

#### 4.2.1. Biosynthesis of Glutathione

Glutathione is a conserved antioxidant in plants, mammals, bacteria and archaea. Moreover, the conjugation of toxic molecules with GSH helps in the detoxification of the cells from dangerous substances ([7,8,9,10]). GSH, the most abundant and available form of thiol in the cell is found in both the cytosol and the organelles, with concentrations ranging between 0.5 and 10 mM [7]. Glutathione exists in reduced (GSH) and oxidized states (GSSG), and the ratio between the two indicates the cellular oxidative stress degree. Typically, cells contain more than 90% of GSH, while the rest is in the disulfide form [7]. In biological reactions, GSH is a cofactor, that donates its hydrogen while generating GSSG. Then, the enzyme glutathione reductase (GSR) converts GSSG to GSH using NADPH/H+ as a cofactor:

NADPH + GSSG + H_2_O → 2 GSH + NADP^+^ + OH^−^ (4)

The biosynthesis of GSH involves two ATP-dependent steps: The first reactions is the synthesis of γ-glutamylcysteine from the Glu and Cys. This reaction is under the control of γ-Glu-Cys ligase (GLC), and it represents the rate-limiting step of GSH synthesis. Besides, it underlines GSH’s importance for life since GLC knockout is lethal [11]. The second reaction is the addition of Gly to the C-terminal γ-glutamylcysteine, and this passage is catalyzed by glutathione synthetase (GSS).

#### 4.2.2. Uptake of Cysteine

A balanced diet plan provides the correct amount of Cys required for GSH biosynthesis [11]. In the extracellular environment, Cys and cystine (cysteine dipeptide, Cys2) are present as GSH and GSSG, extruded from the cells via the multidrug resistance-associated proteins (MRP/ABCC), expressed under the control of nuclear factor erythroid 2-related factor (Nrf2) [11]. Once outside the cell, both Cys and GSH are oxidized to GSSG and Cys2.

**Figure 2:**
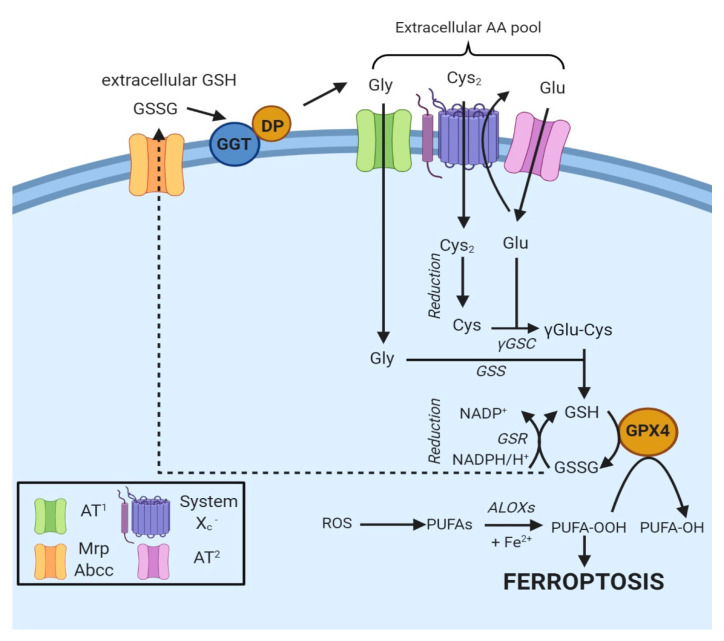
Oxidized GSH (GSSG) is exported from the cell via the MRP/ABCC transporter and hydrolyzed by GGT and dipeptidase to Gly, Glu and Cys_2_, thus contributing to the extracellular amino γ-glutamylcysteine acid pool. Specific amino acid transporters (AT1 and AT2) internalize Gly and Glu, while Cys_2_ is taken up by system X_c_^−^ [11]. Once in the cell, Cys_2_ is reduced to Cys and combined with Glu to generate γGlu-Cys [11]. The addition of Gly to the dipeptide catalyzes the formation of GSH, a cofactor of GPX4. The box on the bottom left indicates the proteins involved.

In most cells, ubiquitously expressed and rather nonspecific neutral amino acid transporters internalize Cys [12]. Besides, cells express two specific Cys transporters: system b^0+^ and system X_c_^−^. The first one is exclusively expressed in kidneys [7]. In contrast, system X_c_^−^ is constitutively expressed in various organs (e.g., brain and immune system) or induced in others in the presence of oxidants or electrophiles [12]. Cystine is internalized by the cells after the reduction of Cys with unknown modalities. At the same time, GSH or GSSG are hydrolyzed by γ-glutamyl transpeptidase (GGT), an enzyme facing the outer layer of the membrane (Figure 2) [11]. The hydrolysis produces di- or tetrapeptides containing Gly and Cys or two Gly and Cys2, allowing the recycling of cysteine.

Finally, some other amino acids or peptide transporters exist, which complicate the general scheme. Of note, in 2015, Newstead proposed the existence of proton-coupled oligopeptide transporter family members (PEPT1 and PEPT2) at the extracellular membrane in mammalian cells. These transporters internalize the Cys-Gly dipeptide generated after the dissociation of GSSG by GGT [13].

#### 4.2.3. System X_c_^−^

The impact of GSH levels on ferroptosis emerged from the use of erastin, a compound that decreases the intracellular concentration of GSH by targeting the cystine/glutamate antiporter system X_c_^−^ [2]. In system X_c_^−^, sodium-independent antiporter imports cystine and exports glutamate with a ratio 1:1 in an ATP-dependent manner [2]. Inhibition of system X_c_^−^ determines a decrease in GSH levels and the beginning of ferroptosis. The system was initially characterized in human fetal lung fibroblasts in 1980 [14]. Then, it became clear that the induction of the antiporter was tissue-specific, and that it occurred in several organs such as lung, kidney and liver, under conditions of oxidative stress [12].

Once internalized, cystine is rapidly reduced to cysteine, the limiting precursor for glutathione GSH synthesis [11]. To assure the import of Cys_2_, intracellular level of glutamate is maintained high so that it can be easily exported. Increasing the concentration of extracellular glutamate might be a solid competitive inhibition strategy to prevent cystine uptake and induce ferroptosis.

The system X_c_^−^ structure consists of two subunits, the 4F2 heavy chain (4F2hc/CD98/SLC3A2) and a light chain (xCT/SCL7A11). The latter subunit exhibits homology with the light chains of heterodimeric amino acid transporters (HATs), a family of amino acid transporters formed by a light and a heavy chain linked by a disulfide bridge [15].

#### 4.2.4. Cysteine Synthesis via the Transsulfuration Pathway

The transsulfuration pathway, in conjunction with methionine metabolism, metabolically produces Cys. Methionine can be converted to Cys to fulfill the requirement in some organs under normal physiological conditions [7]. Methionine is first converted to homocysteine, which is transformed into cystathionine and finally to Cys. In this peculiar context, the carbon backbone of Cys derives from serine, while cystathionine serves as a sulfur donor [7].

This pathway helps to maintain the supply of Cys in some organs, notably in the liver, thus preventing ferroptosis from arising after inhibition of system X_c_^−^. Primary hepatocytes can survive for several days without Cys or cystine in culture media, thanks to the protective role of this pathway [16]. When cysteinyl-tRNA synthetase is knocked down, the transsulfuration pathway is activated, and this leads to the inhibition of Cys-deprivation ferroptosis [17]. This result underlines a connection between protein synthesis and Cys metabolism. It is also necessary to mention that Cys can derive from intra-lysosomal protein degradation. After proteolysis, Cys_2_ is released into the cytosol via cystinosin, H^+^-driven lysosomal transporter (SLC66A4) and it becomes available for GSH synthesis. In humans, a defect of cystinosin leads to rare hereditary disease cystosinosis, caused by crystallization of Cys_2_ in the lysosomes [7,8,9,10].

### 4.3. The Enzyme Glutathione Peroxidase 4 and Ferroptosis

The O_2_-dependent oxidation of carbon fuels in the mitochondria allows for the generation of abundant ATP, but gives rise to partially reduced oxygen species [18]. ROS are generated by mitochondria as well as by enzymes involved in lipid metabolisms such as lipoxygenases, cyclooxygenase, cytochrome P450 and NADPH oxidases [8,9,10]. Oxidation of cholesterol and phospholipids containing PUFAs by lipoxygenases can lead to lipid peroxidation, membrane damage and ultimately cell death [18]. Lipid hydroperoxides (R-OOH) can modify membrane structure and function. Moreover, these lipid hydroperoxides are unstable, and, in the presence of redox-active iron, their structures can change generating small-molecule reactive electrophiles, which lead to new free radical reactions [8,9,10]. The rupture of fatty acids backbone can also lead to the formation of reactive aldehydes such as 4-hydroxy-nonenal (4-HNE) and malondialdehyde (MDA), which can attack proteins or DNA, amplifying the cellular damage [8].

To ensure membrane integrity and minimize ROS-incurred damage, lipid hydroperoxide glutathione peroxidase 4 (GPX4) converts lipid hydroperoxides (R-OOH) to lipid alcohols (R-OH) using reduced GSH as a cofactor [8]. This process prevents the iron (Fe^2+^)-dependent formation and accumulation of toxic lipid ROS, more toxic than cytosolic ROS in ferroptosis [18]. However, lipids such as eicosanoids, derived from enzymatically oxygenated PUFAs, play a significant signaling role, especially in mediating inflammation. Thus, GPX4 has a double function in cellular homeostasis: as a guardian against oxidative damage as well as the physiological regulator.

For this reason, GPX4 seems to be vital for development. Indeed, *GPX4^−/−^* mouse embryos die right after implantation at Day 7.5. GPX4 is defined as the master regulator of ferroptosis, and its direct or indirect inhibition with small compounds leads to ferroptosis [8,9,10,11,12,13,14,15,16,17,18,19].

From a structural point of view, GPX4 is a monomeric enzyme with a molecular size smaller than the subunits of the other GPXs, and this structure affords a broader substrate specificity than other glutathione peroxidases [20]; this allows GPX4 to react directly with LOOHs in membranes with high efficiency.

The pharmacological inhibition of GPX4 triggers the ferroptotic process by the accumulation of lipid peroxides. On the one hand, the induction of ferroptosis may help get rid of therapy-resistant cancer cells, thus enhancing the effects of chemotherapy or radiotherapy and opening up new therapeutic fields [8]. On the other hand, GPX4 could be neuroprotective, and increasing GPX4 function could be a therapeutic strategy for neurodegenerative diseases [8,9,10].

### 4.4. Lipid Metabolism and Ferroptosis

Oxidative damage of membrane lipids is central to the execution of ferroptosis. Peroxidation can occur on both free PUFAs and PUFA-containing membrane phospholipids (PUFA-PLs) (e.g., phosphatidylethanolamine, phosphatidylcholine and cardiolipin). Treatments with different ferroptosis inducers produce a different type of oxidized PL species, and only some of these oxidized species contribute directly to cell death [21]. PUFA-containing phosphatidylethanolamines (PEs) are more susceptible to peroxidation than PUFAs with phosphatidylcholine (PC), because of PEs’ molecular geometry. PEs assume a more conical shape due to the smaller size of the PE head-group. Peroxidation can develop through enzymatic reactions or non-enzymatic processes. In particular, lipoxygenases are the enzymes most involved in the generation of lipid peroxides. The second model affirms that the majority of lipid peroxidation arises due to the spontaneous, non-catalytic process of autoxidation with Fenton chemistry [10,11,12,13,14,15,16,17,18,19,20,21,22].

#### 4.4.1. Role of Lipoxygenases

The formation of lipid peroxides of arachidonic acid (AA) and other ω-6 fatty acids containing PE requires three enzymes: arachidonate lipoxygenases (ALOXs), acyl-CoA synthetase long-chain family 4 (ACSL4) and lysophosphatidylcholine acyltransferase 3 (LPCAT3) [23]. ACSL4 catalyzes the formation of AA-CoA, then LPCAT3 controls the esterification of AA-CoA into AA-PE, and the final oxidation of AA-PE to AA-OOH-PE is run by ALOXs.

The ACSL proteins are mainly localized at the endoplasmic reticulum (ER) and at the outer mitochondrial membrane (OMM). ACSLs are responsible for the formation of fatty acyl-CoA esters from free long-chain fatty acids. There are five isoforms of ACSLs (ACSL1, -3, -4, -5 and -6), but only ACSL4 correlates with ferroptosis, and it is considered the marker of ferroptosis sensitivity. GPX4^−/−^ leads to ferroptotic cell death, while GPX4^−/−^ and ACSL4^−/−^ double knockout cells survive and usually grow [24]. ALOXs are crucial regulators of enzymatic lipid peroxidation. ALOX enzymes are non-heme iron-containing dioxygenases that oxidize PUFAs in a regio-, stereo-, and enantio-specific manner [22]. They catalyze the insertion of oxygen in a molecule of arachidonic acid to generate lipid peroxides. ALOX enzymes prefer free PUFAs as substrates for oxidation. Because of this, phospholipases are likely associated with LOX in cleaving the PUFA acyl chain from PLs. The mechanisms by which LOXs drive ferroptotic cell death and the isoforms that drive this process remain elusive [25].

#### 4.4.2. Lipid Peroxidation

Non-enzymatic lipid autoxidation accounts for three main steps. In membranes, initiation is operated by electron transfer from Fe^2+^ to LOOH through the Fenton reaction, producing the alkoxy radical (LO^•^). In particular, Fenton chemistry refers to a series of reactions between LOOHs and Fe^2+^ to produce oxygen radicals (Section 4.1). In physiological conditions, cellular iron is complexed with ferritin or with heme proteins, and only a small amount of iron remains free, soluble and chelatable. The latter is called the labile iron pool (LIP), and it represents the source of iron for the Fenton reaction.

LOOH + Fe^2+^ → LO^•^ + Fe^3+^ + H_2_O (5)

At this point, LO^•^ interacts with a neighboring lipid collecting an H (L’H), thus forming a carbon-centered radical (L’ ^•^) and then L’ ^•^ reversibly adds oxygen to form a lipid hydroperoxyl radical (L’OO^•^). The creation of new lipid radicals represents the second step (*propagation*).

LO^•^ + L’H → LOH + L’ (6)

L’^•^ + O_2_ ← → L’OO (7)

From LOO^•^, LOOH is generated by H transfer, which stabilizes the reversible oxygen addition. When H is donated by a CH_2_ carbon of an unsaturated fatty acid esterified in a complex lipid, the newly formed L’ ^•^ adds oxygen, thereby propagating lipid peroxidation.

LOO^•^ + L’H → LOOH + L’ (8)

The final step is the termination of the process. It happens either in the presence of antioxidants or by another radical PUFA. LOO^•^ can be stabilized by H donors such as tocopherols or coenzymes Q, which act as antioxidants (Aox-OH). This reaction is called “chain-breaking” because Aox-O^•^ is not able to propagate the peroxidative chain reaction. In the presence of Fe^2+^, LOOH reacts with the reduced transition metal, and the peroxidation-initiating species LO^•^ is produced (Reaction 1).

LOO^•^ + Aox-OH → LOOH + Aox-O (9)

In the presence of a large amount of LOO^•^, radicals react together to form stable products and arrest the reaction. A hydroxyl and a keto derivative of the fatty acid chain (LOH and L = O) are generated, while oxygen is released in an electronically excited state that decays while emitting light.

2 LOO^•^ → LOH + L = O + O_2_ (10)

#### 4.4.3. Where Does Lipid Peroxidation Take Place?

Lipid peroxidation affects all cellular membranes. It affects the lipid bilayer and the subcellular membranes of mitochondria, endoplasmic reticulum (ER) and lysosomes. Mitochondria might promote ferroptosis under specific conditions. Mitochondrial respiration may contribute to the overall pool of ROS in the cell. However, these organelles are not essential for ferroptosis, because cells deprived of mitochondria are exposed to ferroptosis [25]. However, mitochondrial membranes may also be oxidized during ferroptosis; although this oxidation does not appear to be required for death, the accumulation of LOOHs at the mitochondrial membranes could increase permeability, thus explaining mitochondrial swelling [2] and outer membrane rupture [26]. The ER is the first intracellular site of de novo lipid synthesis; thus, lipid peroxidation might happen here. A possible association between ER and ferroptosis could be evaluated, but the onset of lipid peroxidation in the ER and its relationship to ferroptosis is still unclear [25].

Finally, lysosomes can be engaged in ferroptosis. Indeed, ferrostatins, which are ferroptosis inhibitors, localize at the lysosomes, and structural modifications reduce their trapping by the lysosomes, thus increasing their efficacy in suppressing ferroptosis [27]. Moreover, the discovery of ironomycin, an iron-dependent lethal compound localized at the lysosomes, suggests that additional studies concerning the role of lysosomes in ferroptosis are needed [25].

The role of lipid peroxidation is undisputed, but it remains unclear how lipid peroxidation exerts a toxic effect on the cell and leads to cell death. At least three hypotheses have been proposed to answer these questions [22]: (i) LOOH accumulation could modify membrane integrity and alter its biophysical proprieties; (ii) LOOHs may change the localization or function of membrane-associated proteins; and (iii) degradation of LOOHs into highly reactive products may help in the permeabilization of membranes, and this could be directly toxic.

Lipid peroxidation disrupts ion gradients, decreases membrane fluidity, slows lateral diffusion and increases membrane permeability [25]. Moreover, the formation of protein-based pores during ferroptosis has been hypothesized, leading to loss of ionic homeostasis [28].

Lipid peroxidation of PUFAs generates different oxidation products, some of them highly cytotoxic and pathogenic. The best known are MDA and 4-HNE. MDA is obtained from the decomposition of AAs and larger PUFAs via enzymatic and non-enzymatic pathways [25]. MDA reacts with proteins and DNA to form crosslinked adducts. Overload of MDA is associated with many human diseases such as Alzheimer’s and Parkinson’s diseases, cancer, cardiovascular diseases and diabetes [22]. The following product generated is 4-HNE, an electrophile compound, widely studied as a signaling molecule, known for its ability to stimulate the cell cycle and cell proliferation and as a cytotoxic molecule. In particular, 4-HNE can inhibit gene expression and promote the onset of diseases.

### 4.5. Iron Metabolism and Ferroptosis

Iron homeostasis is strictly controlled at the cellular and systemic levels to provide the right amounts of iron needed for the vital biological functions of cells and tissues, avoiding iron overload and iron-related toxicity [29]. Iron trafficking is an example of a circular economy [30]; the human body’s daily iron requirement is around 20–25 mg, which is mainly used in erythropoiesis. A balanced diet provides about 14 mg of iron as inorganic or organic iron [29]. In an adult at a steady-state, 1–2 mg iron is absorbed daily in the gut and compensates for an equal loss (bleeding and desquamation). Since an insufficient amount of iron is absorbed, most of the iron is recycled from the phagocytosis of senescent red blood cells by macrophages.

In our body, iron is found in both ferrous (Fe^2+^) and ferric (Fe^3+^) forms; it is used for the synthesis of metalloproteins, to form organic (e.g., heme) or inorganic cofactors (e.g., Fe/S clusters) [31].

Heme and non-heme iron from dietary sources exhibit different absorption, once they arrive in the gut. Heme carrier protein-1 (HCP-1) internalizes iron in the heme form, but not the non-heme form. Heme is rapidly catabolized by heme oxygenase 1 (HO-1), and iron is released [31].

Inorganic iron is found in the gut in its ferric form. Duodenal cytochrome B reductase (DCYTB) reduces the ferric into the ferrous form, and then the apical divalent metal transporter 1 (DMT1) imports Fe^2+^. At the basolateral membrane of enterocytes, ferroportin (FPN), the principal iron exporter, transports Fe^2+^. At the basolateral side, Fe^2+^ is oxidized to Fe^3+^ by membrane ferroperoxidase (hephaestin or ceruloplasmin) before being captured by transferrin (Tf) in the plasma. Hepatocytes produce Tf, which distributes iron to all organs, including the sites of use (bone marrow) and storage (liver) [31]. Binding to its ubiquitous transferrin receptor protein-1 (TRF1), transferrin delivers iron to cells through the endosomal cycle: the Fe-Tf/TRF1 complex is internalized by endocytosis, and iron is released from Tf in the acidic endosomes. Here, the six-transmembrane epithelial antigen of prostate 3 metalloreductase (STEAP3) reduces Fe^3+^, thus enabling the endosomal DMT1 to export iron. At this point, the acquired iron can be stored in ferritin or transported by FPN to maintain the labile iron pool at a low level to avoid cell toxicity. Excess cellular iron promotes lipid peroxidation through the Fenton reaction and production of ROS, which then triggers ferroptosis. Several studies have demonstrated the connection between iron metabolism genes and increased sensitivity to ferroptosis. Silencing of *TRFC*, the gene encoding TRF1, can inhibit erastin-induced ferroptosis, preventing the accumulation of LIP, while HO-1 accelerates the ferroptotic process by supplementing iron [32].

Furthermore, shRNA-mediated silencing of iron-responsive element-binding protein 2 (IREB2) alters the expression of many iron genes such as *TRFC*, *FTH1* and *FTL*, thereby modifying iron uptake, metabolism and storage [2]. In 2015, Sun et al., described the potential involvement of heat shock proteins (HSPs) in ferroptosis. They demonstrated that heat shock factor-binding protein 1 (HSPB1) was highly inducible after treatment with erastin in several types of cancer cells. In HeLa cells, phosphorylation of protein kinase C (PKC) activates HSBP1. Once activated, HSBP1 reduces iron levels by inhibiting TRF1 expression [33]. In summary, phosphorylated HSPB1 acts as a negative regulator of ferroptosis, reducing the uptake of iron and lipid peroxidation, while inhibition of HSBP1 increases erastin-mediated ferroptosis both in vivo and in vitro [33].

The role of iron in ferroptosis is now established, but the connection between the inorganic metal and the biological process remains unclear. The development of new fluorescent probes to detect LIP levels in various cells or organs in live animals could help improve understanding of the role of iron in ferroptosis in multiple conditions. In 2019, Hirayama et al., managed to establish an imaging method to monitor organelle-specific labile Fe^2+^, in mitochondria, lysosomes and endoplasmic reticulum at the same time during erastin-mediated ferroptosis [34].

### 4.6. p53-Mediated Ferroptosis

p53 is a tumor suppressor defined as the “guardian of the genome” because of its role in preserving the stability of the DNA by preventing mutations. p53 works as an antitumor protein with precise functions in apoptosis, necrosis and autophagy. p53 antitumoral activity is due to its DNA-binding transcriptional factor role. [23]. In 2012, Li et al., showed that posttranslational modifications of p53 have a substantial impact on its transcriptional and oncosuppressive functions. The acetylation-defective mutant referred to as p53^3KR^ fails to induce apoptosis and cell cycle arrest; however, the tumor suppression capacity is retained by p53^3KR^ as a wild-type p53, suggesting an additional pathway that mediates tumor development. One target p53^3KR^ is *SLC7A11*, a gene encoding for the subunit xCT/SCL7A11 of system X_c_^−^. The binding downregulates the expression of *SLC7A11*, thereby affecting the activity of GPX4. The transcriptional repression of *SCL7A11* results in the reduction of antioxidant capacity, ROS accumulation and ferroptosis instauration [3]. p53^4KR98^, a genetic variant with four mutations of acetylated lysine residues, loses tumor suppression activity [35]. Therefore, acetylation of the residue Lysine 98 is essential for p53-mediated ferroptosis [23]. Genetic inactivation of SCL7A11 and inhibition of system X_c_^−^ impair cystine uptake and GSH synthesis, causing lipid peroxide accumulation and ferroptosis.

p53 is also involved in polyamine metabolism, which impacts cellular growth, development, and programmed cell death [35]. p53 can also negatively regulate ferroptosis: this anti-ferroptosis regulator activity depends on p53 protein stabilization and *trans*-activation of CDKN1A/p21 (cyclin-dependent kinase inhibitor 1A), responsible for cycle cell arrest. Cell treatment with nutlin-3 (inhibitor of MDM2/p53 interaction) increases p53 expression and stability and, at the same time, inhibits erastin-induced ferroptosis in p53^+/+^ cells, but not in p53^−/−^ cells [10]. Cells treated before with nutlin-3 and then with erastin show a delay in the onset of ferroptosis. Moreover, nutlin-3 upregulates CDKN1A mRNA and p21 protein in p53^+/+^ cells, but not in p53^−/−^ cells [10,11,12,13,14,15,16,17,18,19,20,21,22,23,24,25,26,27,28,29,30,31,32,33,34,35]. The p53/p21 axis delays the execution of ferroptosis by reducing the consumption of GSH, but it is not clear how p21 promotes GSH biosynthesis. In colorectal cancer (CRC), the translocation of dipeptidyl-peptidase 4 (DPP4) by p53 to the nucleus prevents the onset of ferroptosis. Moreover, p53 promotes the expression of *SCL7A11* in CRC cells, but, at the same time, it inhibits the expression of the same in other tumor cells (U2OS and MCF7 cells). It is possible to conclude that p53 plays dual regulatory functions in ferroptosis regarding its target genes (*SCL7A11*, *SAT1* and *CDKN1A/p21*), and its role could depend on tumor cell type [23].

### 4.7. Ferroptosis and the Keap1–Nrf2 Pathway

The nuclear factor erythroid 2-related factor (Nrf2) is a crucial regulator of the antioxidant response [36] and the inducible cell defense system [37]. It is a basic leucine zipper (bZIP) transcription factor, and it heterodimerizes with its partners, small Maf proteins [37]. Under physiological conditions, Kelch-like ECH-associated protein 1 (Keap1) helps maintain low levels of Nrf2, stimulating its ubiquitination and proteasomal degradation. Conversely, during oxidative stress, Nrf2 protein is derepressed and stabilized, and it initiates a multistep pathway activation [36]. This process is strictly under the control of p62, an autophagy receptor, a multifunctional protein, which inhibits Keap1 directly and promotes Nrf2 activation simultaneously. In 2016, Sun et al., demonstrated that the p62-Keap1-Nrf2 pathway inhibits ferroptosis in hepatocellular carcinoma (HCC) [36].

Nrf2 translocates to the nucleus, it binds to antioxidant response elements (AREs) located in the regulatory regions and activates transcription of cytoprotective genes while undergoing heterodimerization. Nrf2 activates multiple cell defense mechanisms, thereby potentiating the detoxification activity of cells [37]. Nrf2 regulates a plethora of targets such as genes involved in the regulation of synthesis and conjugation of GSH (e.g., *GCL, GSR*, etc.) or genes encoding antioxidant proteins (e.g., thioredoxin (*TXN*) and thioredoxin reductase 1 (*TXNRD1*)). Heme oxygenase 1 (-*HO-1*), ferrochelatase (*FECH*) and both ferritin heavy and light chains are strictly under the control of Nrf2, as well.

*Nrf2^−/−^* mice are more susceptible to drug-induced toxicity and oxidative stress [38]. Therefore, the enhancement of Nrf2 expression through pharmacological or genetic approaches could reduce the level of ROS, avoiding the intensification of stress-induced diseases (e.g., acute lung injury, diabetic nephropathy, heart failure and cancer) [37]. However, the activation of Nrf2 shows considerable side effects, since an elevated activity of Nrf2 decreases the therapeutic response of cancer cells to radiotherapy and chemotherapeutic treatments. Thanks to this, cancer cells increase their capacity for detoxification of dangerous secondary products (e.g., ROS) and can generate continuously [39]. Several lines of evidence of Nrf2′s double role are progressively accumulating in the literature. For instance, in 2020, Liu et al., demonstrated that prolonged administration of erastin induced ferroptosis resistance in ovarian cancer cells [40]. They observed a sustained upregulation of cystathionine-β-synthase (CBS), which converts homocysteine into cystathionine in the transsulfuration pathway (see Section 4.2.4). In addition, Nrf2 was found to be constitutively activated and positively correlated with CBS induction. In conclusion, the activation of Nrf2/CBS accounted for ferroptosis resistance in ovarian cancer cells [40]. However, Nrf2 guards cells against stress-induced diseases, such as ischemia–reperfusion (I-R) injury. The protective role of Nrf2 was evaluated in renal proximal tubular epithelial cells (RPTECs) of the native hibernator, the Syrian hamster [41]. The reoxygenation enhanced the expression of Nrf2, leading to the upregulation of antioxidant enzymes (e.g., superoxide dismutase 3 (SOD3) and GSR) and of anti-ferroptotic proteins (FTH1/FTL) in hamster RPTECs, rescuing cells from reoxygenation-induced cell death [41].

### 4.8. Other Related Signaling Pathways

Other cellular pathways may trigger ferroptosis. Ferroptosis suppressor protein 1 (FSP1) is a potent resistance factor and acts as a potent ferroptosis suppressor in vitro and in vivo. FSP1 contains a short N-terminal hydrophobic sequence and a FAD-dependent oxidoreductase domain [42]. The N-terminal consensus sequence is subjected to myristoylation, a fatty acid modification that mediates FSP1 recruitment to the plasma membrane. Here, FSP1 acts as an oxidoreductase reducing coenzyme Q10 (CoQ10) and generating a lipophilic radical-trapping antioxidant (RTA) that halts the propagation of lipid peroxides [42]. FSP1 could provide a new target for the development of drugs targeting the inhibition of ferroptosis [43].

Currently, the role of mitochondria in ferroptosis remains controversial. During ferroptosis, mitochondria undergo morphological changes, including outer membrane fragmentation and cristae enlargement. Furthermore, mitochondrial voltage-dependent anion channels (VDACs) are targets of erastin, and their inhibition induces ferroptosis in human tumor cells. Additionally, mitochondria have a central role in oxidative metabolism, which is indispensable for ferroptosis. In 2019, Gao et al., demonstrated that the mitochondrion is a crucial and proactive player in cysteine deprivation-induced ferroptosis, but not in GPX4 inhibition-induced ferroptosis [44]. They also showed that the TCA cycle and electron transport chain promote ferroptosis, acting as a significant source of lipid ROS. In a recent study, the role of CDGSH iron sulfur domain 1 (CISD1, also termed mitoNEET) was investigated. CSD1 is an iron-containing outer mitochondrial membrane protein. Functionally, it regulates the mitochondrial iron uptake and respiratory capacity. The genetic inactivation of CSD1 increases iron-mediated intramitochondrial lipid peroxidation, which contributes to erastin-induced ferroptosis [45].

## 5. Crosstalk between Autophagy and Ferroptosis

Autophagy is an evolutionarily conserved catabolic process that allows lysosomes to degrade cytoplasmic components (e.g., unused proteins, damaged organelles and pathogens). Various stresses can stimulate autophagy, and excessive degradation of cytosolic components can lead to the triggering of ferroptosis. In particular, some selective autophagy, such as ferritinophagy, contribute to iron accumulation and free radical damage during ferroptosis. Ferritin, a globular protein, is the major iron storage protein (~4500 Fe atoms) [46].

Ferritin is composed of two distinct ferritin subunits, the heavy (FTH1) and ferritin light chain (FTL). The enzymatic activity of the FTH1 subunit rapidly oxidizes Fe^2+^ to Fe^3+^, and it incorporates iron into the shell [46].

Ferritin depletion results in an iron release, which increases the labile iron pool and subsequent cellular toxicity. Consequently, ferritinophagy plays an essential role in maintaining iron homeostasis. Ferritin can be degraded by two mechanisms: lysosomes and proteasomes [47]. The degradation of ferritin via lysosome was discovered in 2014 by Mancias’s team. They identified nuclear receptor coactivator 4 (NCOA4), which is a specific cargo receptor for the autophagic degradation of ferritin [48]. Under iron-depleted conditions, NCOA4 binds the iron-rich ferritin in the autophagosome and delivers it to the lysosome for iron release [46]. The binding between NCOA4 and ferritin is the FTH1-specific. In the presence of a high concentration of iron, NCOA4 is ubiquitinated by ubiquitin ligase, HERC2, and degraded, affecting protein stability [47]. NCOA4-deficient cells fail to induce ferritinophagy, and they are associated with decreased bioavailability of iron. The initiation of ferroptosis activates ferritinophagy to increase the labile iron pool (LIP), which promotes ROS accumulation, which drives the ferroptotic process. Therefore, inhibition of NCOA4 represses ferritin degradation and suppresses ferroptosis, while its overexpression has the opposite effects [47].

## 6. Pharmacological Modulation of Ferroptosis

The ferroptosis pathway can be either triggered using small exogenic compounds or by modulating physiological conditions (e.g., high/low concentration of extracellular glutamate) [49]. Ferroptosis chemical inducers were discovered before the biological process.

### 6.1. Ferroptosis Inducers

**Figure 3:**
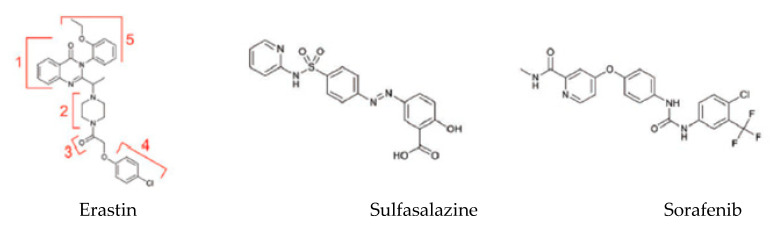
Exogenous ferroptosis inducers [49].

A variety of different substances can lead to the induction of ferroptosis with diverse cellular targets [3] (Table 2). The first category includes erastin, sulfasalazine and sorafenib (Figure 3). These three small molecules bind the same transporter system X_c_^−^.

Erastin is the prototype of the inhibitor, which directly targets system X_c_^−^. Structural analysis of the molecule demonstrates that the quinazolinone scaffold (Part 1) ensures drug lethality while increasing the flexibility of the piperazine linker (Part 2) would decrease drug activity [49]. Moreover, modifications of Parts 3 and 4 would reduce or even eliminate the inhibitory ability of erastin (Figure 3) [49]. Only the chlorine atom of erastin is designated as a binding site with the cellular environment. Additional groups (e.g., bromo, phenyl or furanyl groups) on Part 5 can improve the effectiveness of erastin at its target [49]. VDAC is directly inhibited by erastin, and this can lead to mitochondrial dysfunction. VDAC 2/3 are outer mitochondrial membrane channels, and their inhibition leads to the rupture of the OMM. Moreover, there are pieces of scientific evidence suggesting that the cellular knockout of VDAC2 and VDAC3, but not VDAC1, leads to erastin resistance [5].

Sulfasalazine (SAS) is broadly used as a first-line anti-inflammatory drug for the treatment of rheumatoid arthritis [49]. SAS induces ferroptosis by inhibiting system X_c_^−^ similar to erastin, but less potently.

Sorafenib is a clinically approved multikinase inhibitor for the treatment of advanced carcinoma (e.g., renal cell carcinoma, hepatocellular carcinoma and thyroid carcinoma) [49]. In some cancer cell lines, drug resistance has been described after treatment with sorafenib: it has been demonstrated that upregulation of Nrf2 and pRB can inhibit sorafenib-induced ferroptosis in hepatocellular carcinoma cell lines [50].

After the discovery of erastin, other inducers were found thanks to high-throughput screening. Ras-selective lethal small molecules (RSL3 and RSL5) are included in this second category. These molecules require the presence of iron, ROS and MEK in an activated state to trigger ferroptosis in Ras-mutated tumor cells. [49]. RSL3 can directly bind and inactivate GPX4, increasing the production of lipid ROS. RSL3 binds enzymes with a nucleophilic site (e.g., cysteine, serine, selenocysteine, etc.). RLS3 inactivates GPX4-mediated alkylation of the selenocysteine present at the reactive site [49].

The third category of ferroptosis inducers includes FIN56. Two different pathways contribute to the induction of ferroptosis by FIN56. First, FIN56 promotes the degradation of GPX4, which requires the catalysis of the enzyme acetyl-CoA carboxylase (ACC). Second, FIN56 binds to and supports the activation of the enzyme squalene synthase [49]. This stimulation leads to the depletion of the endogenous antioxidant coenzyme Q10, which is essential for preventing the accumulation of lipid ROS, and it enhances the sensitivity of the cell to ferroptosis.

The last category includes FINO2, 1,2-dioxolane, which causes cell death in two ways: (i) the direct oxidation of LIP; and (ii) the inactivation of GPX4 [49].

### 6.2. Ferroptosis Inhibitors

With the increasing relevance of the ferroptosis pathway as a pharmacological target, strategies aiming at the inhibition of lipid peroxidation have been developed over the years. Since lipid peroxidation can occur via enzymatic and non-enzymatic pathways, ferroptosis inhibitors can be divided into two major groups: lipid autoxidation inhibitors (e.g., radical trapping antioxidant) and lipoxygenase inhibitors.

The lipoxygenase inhibitors group consists of several different compounds, including iron chelators, which can inactivate the enzyme by removal of the active site iron [51]. RTAs are compounds that react with chain-carrying radicals, interrupting the autoxidation chain reaction. This category includes α-tocopherol, the most biologically active form of vitamin E, ferrostatins and liproxstatins.

High-throughput screening of small molecule libraries brought to the identification of two potent ferroptosis inhibitors: ferrostatin-1 (Fer-1) and liproxstatin-1 (Lip-1) [5]. In this screening assay, knockout for the gene *GPX4* or pharmacological inhibition of system X_c_^−^ reproduced the ideal condition for the stimulation of ferroptosis cell death [5].

**Figure 4:**
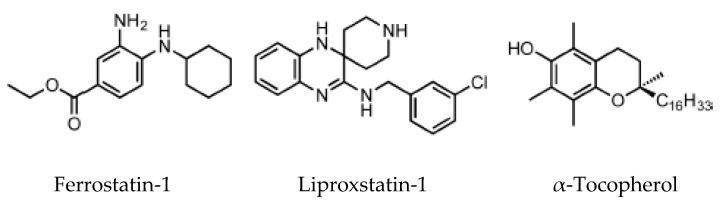
Ferroptosis inhibitors [52].

Ferrostatins inhibit lipid peroxidation associated with erastin- and RSL3-induced ferroptosis. The activity of the first generation of ferrostatin, termed ferrostatin 1, depends on the primary aromatic amine (Figure 4), which specifically reduces the accumulation of lipid ROS. Pharmacokinetics analysis on the second generation (SRS 11-92) and the third generation (SRS 16-86) of ferrostatins showed an increased plasma and metabolic stability of these two compounds, associated with higher protection against tissue injury in vivo [5].

Lip-1 suppresses ferroptosis in the low nanomolar range and demonstrates good pharmacological proprieties, including a half-life of 4.6 h in plasma in a mouse model [51]. Lip-1 decreases VDAC1 protein synthesis, and oligomerization is observed, but the same result has not been found for VDAC 2/3 [51]. VDAC1 is highly permeable to Ca^2+^, and in cardiac ischemia–reperfusion injury (IRI), VDAC1 promotes damage, while VDAC2 may have a protective role [52]. VDAC1 knockdown decreases cell death by reducing the apoptotic Ca^2+^ signal conveyed from the mitochondrial VDAC1 to the endoplasmic reticulum. Therefore, Lip-1 could have a protective role in cardiac IRI by targeting VDAC1. Moreover, Lip-1 plays a role downstream of GPX4, which converts reduced GSH into oxidized GSSH: Lip-1 ameliorates acute renal failure in a genetic GPX4 KO mouse model, strongly suggesting an in vivo anti-ferroptosis activity [51].

## 7. Ferroptosis in Liver Diseases

Hepatocytes play a crucial role in humans by helping maintain stable glucose and lipoprotein concentrations in the plasma. Usually, hepatocytes are quiescent, but a radical change in liver physiology can occur when liver tissue is exposed to viruses, toxic agents or metabolites in excess. Moreover, hepatocytes are the primary site of the storage of iron in the body. Clinicians set 13–15 mg of iron/g of liver tissue as a critical threshold, which is associated with an increased risk of cirrhosis. [29]. The type of liver damage depends on the nature and the severity of the lesion. Different kinds of RCDs may coexist in the progression of metabolic liver diseases to inflammation, fibrosis and, ultimately, cirrhosis [53]. Cirrhosis, a slow process spread over decades, is the most advanced stage of liver fibrosis and is associated with a higher risk of malignant liver transformation into hepatocellular carcinoma (HCC). Accumulating evidence suggests that lytic cell death modalities (e.g., necroptosis, pyroptosis and ferroptosis) elicit strong inflammatory responses due to cell membrane permeabilization and release of cellular components, contributing to the recruitment of immune cells and activation of hepatic stellate cells [53].

The association between liver damage and both inherited and acquired iron overload is indisputable. Ferroptosis could determine iron overload because the induction of ferritinophagy induces the active mobilization of cellular iron. In any case, uncontrolled free iron exerts a toxic effect on the liver, stimulating the advancement of hepatic diseases and leading to severe collateral effects [54].

### 7.1. Ferroptosis and Drug-Induced Liver Injury

Drug-induced liver injury (DILI) is the predominant cause of acute liver diseases (ALD) in Europe and the USA, with acetaminophen as the paradigmatic example [55]. The exposure of hepatic tissue to acetaminophen leads to the hepatocyte cell death [10,11,12,13,14,15,16,17,18,19,20,21,22,23,24,25,26,27,28,29,30,31,32,33,34,35,36,37,38,39,40,41,42,43,44,45,46,47,48,49,50,51,52,53,54,55,56]. Its transformation by cytochrome p450 provokes liver-toxicity through its reactive metabolite NAPQI (*N*-acetyl-p-benzoquinone imine) [56]. NAPQI binds to GSH and leads to severe depletion of GSH in hepatocytes [56]. Yamada’s team has recently found that ferroptosis driven by ω-6 PUFAs is associated with acetaminophen-induced ALD [57]. Besides, ferrostatin-1, DFO and vitamin E could exert a protective effect on hepatocytes by suppressing lipid peroxidation and GSH depletion [57]. Moreover, it was recently shown using CRISPR-Cas9 that cytochrome P450 oxidoreductase, which is directly implied in the detoxification of xenobiotics by hemoprotein, was necessary for ferroptotic cell death by upregulating the PUFAs peroxidation [58].

### 7.2. Ferroptosis and Ischemia–Reperfusion Injury (IRI)

**Figure 5:**
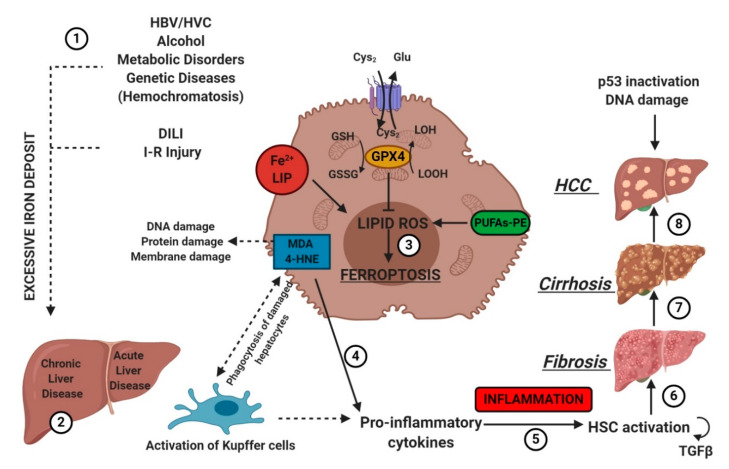
Etiologic factors and the central role of oxidative stress in the development of hepatic diseases. Several factors (e.g., HBV/HCV infection, inadequate alcohol or drug consumption, metabolic or genetic diseases, and I-R injury (1)) may trigger CLD or ALD (2) in the liver, promoting iron accumulation. This supports pro-oxidative state triggering protein and DNA damage and lipid peroxidation (3). The instauration of hepatocyte oxidative stress conditions results in inflammation (4–5), which favors liver fibrosis (6) and cirrhosis (7), and it may lead to hepatocellular carcinoma (8). Numbers (1–8) were added to indicate the path to follow in reading the image. *HSC*, hematopoietic stem cells; *TGFβ*, transforming growth factor-beta.

IRI is the consequence of a temporary reduction of the blood supply followed by revascularization [59]. The restoration of blood supply after ischemia aggravates the pre-existing injury in the liver caused by ischemia. Liver IRI can be induced by shock (e.g., sepsis and hemorrhage) or after liver surgery. In 2014, Friedmann Angeli et al., hypothesized a protective effect of liproxstatin-1 against hepatic damage in a mouse model of hepatic injury induced by IRI [26]. These data evidenced that ferroptosis was a mechanism implied in liver IRI, underlying the potential interest to target ferroptosis in the treatment of IRI (Figure 5).

### 7.3. Chronic Liver Diseases (CLD)

CLD are the consequence of constant exposure of the liver to agents as alcohol, chronic infection such as HBV/HVC or altered intermediary metabolism [10,11,12,13,14,15,16,17,18,19,20,21,22,23,24,25,26,27,28,29,30,31,32,33,34,35,36,37,38,39,40,41,42,43,44,45,46,47,48,49,50,51,52,53,54,55,56]. These conditions induce chronic liver scarring and fibrosis, combined with an increased cell turnover rate.

CLD and iron metabolism are tightly interconnected. Chronic HVC infection favors the accumulation of iron, downregulating hepatic hepcidin and upregulating duodenal ferroportin-1. Progressive iron overload correlates with the worsening of liver damage.

An iron-centered hypothesis has also been proposed in the pathogenesis of the non-alcoholic liver disease (NAFLD) [53]. NAFLD spans from simple hepatic steatosis to non-alcoholic steatohepatitis (NASH), and it is associated with obesity and metabolic syndrome [53] (Figure 5). Steatosis can progress to an inflammatory state known as non-alcoholic steatohepatitis (NASH) and degenerate to fibrosis. This progression correlates with an increase in lipid peroxidation, stemming from the simultaneous presence of fatty acids and a high concentration of iron in hepatocytes [60]. Lipotoxicity, oxidative stress, organelle dysfunction and inflammatory response exacerbate ballooning and increase hepatocyte cell death [61]. The role of ferroptosis in NAFLD and NASH has not been clarified yet, but MDA and 4-HNE have been used as oxidative stress markers in NASH patients [62]. Besides, the hepatic PC/PE ratio is low in NASH patients. Vitamin E suppresses lipid peroxidation and reduces serum transaminases in NASH patients [63]. Conversely, iron overload aggravates NASH [64,65]. Ferroptosis is the first hit event that triggers steatohepatitis and precedes other RCDs. [66]. Ferroptosis inhibitors, as Trolox and deferiprone, suppress cell death and inflammation at the onset of NASH in the choline-deficient, ethionine-supplemented (CDE) diet model. In alcohol liver disease, after ethanol administration, adipose-specific lipin-1 overexpression accelerates iron accumulation, causes lipid peroxidation, reduces GSH and GAPDH and promotes ferroptotic liver damage in mice [67]. Lipin-1 is an Mg^2+^-dependent phosphatidic acid phosphohydrolase, implicated in the formation of diacylglycerol during the synthesis of phospholipids and triglycerides.

Regarding hereditary hemochromatosis, inherited mutations in the *HFE* gene lead to massive accumulation of iron in the liver and chronic tissue damage that may progress to hepatocellular carcinoma. In 2017, Wang’s team induced ferroptosis in the liver by administering ferric citrate to *–SCL7A11* KO mice lacking xCT of system X_c_^−^ [68]. Indeed, *–SCL7A11* KO facilitates iron overload-induced ferroptosis due to impaired cystine uptake and increased ROS production [53,54,55,56,57,58,59,60,61,62,63,64,65,66,67,68]. Ferrostatin-1 reversed the iron-overload damage [68].

In addition, the rupture of the gene encoding Nrf2 stimulates the onset of liver fibrosis in *Hfe^−/−^* by an increased susceptibility to oxidative stress [69]. 

### 7.4. Hepatocellular Carcinoma (HCC)

HCC is the most frequent liver cancer, and it is the second leading cause of cancer-related death worldwide in men [70]. HCC is usually diagnosed late. Sorafenib, a multikinase inhibitor, was the first drug used for the treatment of advanced HCC. Louandre et al., evidenced that sorafenib induces ferroptosis [50]. Since then, sorafenib has been classified as a ferroptosis inducer [71]. Sorafenib exerts its anticancer activity by inducing apoptosis and inhibiting proliferation as well as angiogenesis [71]. It is a weak apoptosis inducer compared to other chemicals, but it produces ferroptosis. Sorafenib-induced ferroptosis can be reversed using either ferrostatin-1 or shRNA. Mechanistically, sorafenib acts as erastin inhibiting SCL7A11, blocking the import of oxidized cysteine into the cell. Many studies have been done to characterize the relationship between sorafenib and cellular pathways. The retinoblastoma protein (Rb) inhibits sorafenib-induced ferroptosis in HCC cells [72]. Therefore, the Rb status of individual HCC patients is an important prognostic marker during treatment with sorafenib.

The p62-Keap1-Nrf2 pathway also plays a central role in the prevention of HCC by acting against sorafenib-induced ferroptosis by upregulating genes (notably *–HO-1* and *FTH1*) involved in iron and ROS metabolism [36]. These genes act as antioxidants, enhancing the resistance to ferroptosis. Another protein involved in sorafenib resistance is the metallothionein-1G (MT-1G), a critical negative regulator of ferroptosis. Its expression is induced by sorafenib and other kinase inhibitors (e.g., erlotinib and gefitinib) [71]. The upregulation of MT-1G contributes to sorafenib resistance. Besides, CISD1 negatively regulates ferroptosis, and it is upregulated in an iron-dependent manner in human HCC cells. Finally, a recent study showed that a variant present in Africans and the Afro-American population in the tumor suppressor *p53* (p.Pro47Ser) downregulates ferroptosis in HCC [71].

Interestingly, Ou et al., reported that low-density lipoprotein-docosahexaenoic acid (LDL-DHA) nanoparticles selectively kill human and rat hepatoma cell lines in vitro and reduce the growth of liver cancer in rat [73]. LDL-DHA exacerbates lipid peroxidation and determines the depletion of GSH and inactivation of GPX4 before cell death. DHA is responsible for the direct degradation of GPX4, while the entire nanoparticle decreases the concentration of intracellular GSH by reducing redox couples GSH/GSSG and NADPH/NADP+ and removing GSH-aldehyde adducts [71]. In 2017, Bai et al., identified haloperidol as a potential pharmacological modulator. Haloperidol, which is antipsychotic medication and a sigma receptor 1 antagonist, was able to promote erastin- and sorafenib-induced cell death at a relatively low dose [74], indicating that haloperidol may benefit HCC patients treated with sorafenib by reducing the dosage or potentiating its effectiveness [71]. After the administration of haloperidol, the distinct traits (iron accumulation, lipid peroxidation and GSH depletion) of ferroptosis were reported; at the same time, haloperidol influenced ferroptosis-related targets (Nrf2, HO-1 and GPX4) [74]. These results provide a novel pharmacological strategy for HCC therapy.

## 8. Conclusion and Perspectives

Nowadays, the idea of ferroptosis-inducing therapy is becoming consistent in the field of cancer treatments. Sorafenib is now the gold standard as a ferroptosis inducer. Moreover, new pharmacological formulations are becoming available for erastin and RSL3. Efforts have been undertaken to render erastin more suitable for in vivo applications, such as erastin-loaded exosomes, to target triple-negative breast cancer [75]. New studies focused on the molecular basis of ferroptosis could help in deciphering the existing connections between the single components of the metabolic pathway. Above all, this could also lead to the identification of new molecular markers for ferroptosis, which are still missing. These markers could also lead to a faster diagnosis in the field of oncology. In this context, ferroptosis may represent the cornerstone for the development of alternative curative approaches.

In summary, new projects dedicated to fundamental research should be encouraged to obtain the full picture of intracellular interactions after ferroptosis induction. Another field of interest is to define the connections between ferroptosis and other cell deaths to identify their co-operation and modulation in cells and tissues. New frontiers are opening, which will put ferroptosis under the spotlight of future translational medicine.

## Figures and Tables

**Table 1:** The main morphological and biochemical features of regulated cell deaths (adapted from Xie et al., 2016 [5] and Lie et al., 2020 [3]). *MAPKs*, mitogen-activated protein kinases; *GSH*, glutathione; *DAMPs*, damage-associated molecular patterns; *PS*, phosphatidylserine; *PARP1*, Poly [(ADP-ribose)] polymerase 1; *LC3-I/II*, Microtubule-associated protein light chain 3.

Cell Death	Morphological Features	Biochemical Features
**Ferroptosis**	No rupture of the plasma membrane [3] Rounding up of the cell [3] Small mitochondria, outer mitochondrial rupture, reduction of the cristae [3] Normal nuclear size and no chromatin condensation [3]	Iron and ROS overload Activation of MAPKs Inhibition of system X_c_^−^ and decreased cystine uptake GSH depletion Release of arachidonic acid mediators [3]
**Apoptosis**	Plasma membrane blebbing [3] Rounding up of the cell [3] Pseudopod retraction and reduction of cellular and nuclear volume [3] Nuclear fragmentation, chromatin condensation Formation of apoptotic bodies [3] No significant changes in mitochondrial structure [3]	Activation of caspases Oligonucleosomal DNA fragmentation PS exposure [3]
**Necroptosis**	Rupture of the plasma membrane [3] Cytoplasmic swelling [3] Moderate chromatin condensation [5] Spillage of cellular constituents into microenvironment [3]	Decrease in ATP level Release DAMPs PARP1 hyperactivation [3]
**Autophagy**	Lack of change in the plasma membrane [3] Accumulation of autophagic vacuoles [3] Lack of chromatin condensation [5] Formation of double-membraned autolysosomes, including macroautophagy, microautophagy and chaperone-mediated autophagy [3]	LC3-I to LC3-II conversion [3] Substrate degradation [5]

**Table 2:** Ferroptosis inducers (adapted from [25]).

Class	Class Characteristics	Impact on Ferroptosis	Compound Examples	Suitable for In Vivo Use
**Class 1**	Inhibition of system X_c_^−^	Prevention of cystine import, GSH depletion, loss of GPX4 activity	Erastin, sulfasalazine, sorafenib	Sorafenib, sulfasalazine
**Class 2**	Direct inhibition of GPX4	Covalent interaction with GPX4 and inhibition of the enzyme	RSL3, RSL5	No
**Class 3**	Depletion of GPX4 protein and CoQ10	Depletion of GPX4 and CoQ10	FIN56	Unknown
**Class 4**	Induction of lipid peroxidation	Oxidation of iron drives lipid peroxidation and indirect inactivation of GPX4	FINO2	Unknown

## Abbreviations

4-HNE	4-hydroxy-noenal
AA	Arachidonic acid
ACC	Acetyl-CoA carboxylase
ACSL4	Acyl-CoA synthetase long-chain family member 4
ALD	Acute liver disease
ALOXs	Arachidonate lipoxygenases
AREs	Antioxidant response elements
ATG5-7	Aumiddlehagy protein 5 and 7
bZIP	Basic leucine zipper
CBS	Cystathionine β-synthase
CDKN1A/p21	Cyclin-dependent kinase inhibitor 1A
CISD1	CDGSH iron sulfur domain 1
CLD	Chronic liver disease
CoQ10	Coenzyme Q10
CRC	Colorectal cancer
DAMPs	Damage-associated molecular patterns
DCYTB	Duodenal cytochrome B reductase
DFO	Deferoxamine
DILI	Drug-induced liver injury
DMT1	Divalent metal transporter 1
DPP4	Dipeptidyl-peptidase 4
ER	Endoplasmic reticulum
Fe^2+^	Ferrous iron
Fe^3+^	Ferric iron
FECH	Ferrochelatase
Fer-1	Ferrostatin-1
FPN	Ferroportin
FSP1	Ferroptosis suppressor protein 1
FTH1	Ferritin heavy chain
FTL	Ferritin light chain
GGT	γ-Glutamyl transpeptidase
GLC	γ-Glu-Cys-ligase
GPX4	Glutathione peroxidase 4
GSR	Glutathione reductase
GSH	Glutathione
GSS	Glutathione synthetase
HATs	Heterodimeric amino acid transporters
HCC	Hepatocellular carcinoma
HCP-1	Heme carrier protein-1
HO-1	Heme oxygenase-1
HSC	Hemamiddleoietic Stem Cell
HSPs	Heat shock proteins
HSPB1	Heat shock factor-binding protein 1
IREB2	Iron-responsive element-binding protein 2
IRI	Ischemia–reperfusion injury
Keap1	Kelch-like ECH-associated protein 1
LC3-I/II	Microtubule-associated protein light chain 3
LIP	Labile iron pool
Lip-1	Liproxstatin-1
LPCAT3	Lysophosphatidylcholine acyltransferase 3
MAPKs	Mitogen-activated protein kinases
MDA	Malondialdehyde
MRP/ABCC	Multidrug resistance-associated proteins
MT-1G	Metallothionein-1G
NAFLD	Non-alcoholic liver disease
NAPQI	N-acetyl-p-benzoquinone imine
NASH	Non-alcoholic steatohepatitis
NCOA4	Nuclear receptor coactivator 4
Nrf2	Nuclear factor erythroid 2-related factor
OMM	Outer mitochondrial membrane
OXPHOS	Oxidative phosphorylation
PARP1	Poly [ADP-ribose] polymerase 1
PC	Phosphatidylcholine
PE	Phosphatidylethanolamine
PEPT1-2	Proton-coupled oligopeptide transporter family members 1 and 2
PKC	Protein kinase C
PS	Phosphatidylserine
PUFAs	Polyunsaturated fatty chains
Rb	Retinoblastoma
RCD	Regulated cell death
ROS	Reactive oxygen species
RPTECs	Renal proximal tubular epithelial cells
RSL3/5	Ras-selective lethal small molecule 3/5
RTA	Radical-trapping antioxidant
SAS	Sulfasalazine
SAT1	Spermidine/spermine N1-acetyltransferase 1
SOD3	Superoxide dismutase 3
STEAP3	Six-transmembrane epithelial antigen of prostate 3
Tf	Transferrin
TGFβ	Transforming growth factor beta
TRF1	Transferrin receptor protein 1
TXN	Thioredoxin
TXNRD1	Thioredoxin reductase 1
VDACs	Voltage-dependent anion channels

## References

[1] Doll S., Conrad M. (2017). Iron and ferroptosis: A still ill-defined liaison: Iron and Ferroptosis’Ferroptosis’. IUBMB Life.

[2] Dixon S.J., Lemberg K.M., Lamprecht M.R., Skouta R., Zaitsev E.M., Gleason C.E., Patel D.N., Bauer A.J., Cantley A.M., Yang W.S. (2012). Ferroptosis: An Iron-Dependent Form of Nonapoptotic Cell ‘Death’. Cell.

[3] Li J., Cao F., Yin H.-L., Huang Z.-J., Lin Z.-T., Mao N., Sun B., Wang G. (2020). Ferroptosis: Past, present and future. Cell Death Dis..

[4] Dolma S., Lessnick S.L., Hahn W.C., Stockwell B.R. (2003). Identification of genotype-selective antitumor agents using synthetic lethal chemical screening in engineered human tumor cells. Cancer Cell.

[5] Xie Y., Hou W., Song X., Yu Y., Huang J., Sun X., Kang R., Tang D. (2016). Ferroptosis: Process and function. Cell Death Differ..

[6] Wang H., Liu C., Zhao Y., Gao G. (2020). Mitochondria regulation in ferroptosis. Eur. J. Cell Biol..

[7] Fujii J., Kobayashi S., Homma T., Tang D. (2019). Regulation of Ferroptosis Through the Cysteine-Glutathione Redox Axis. Ferroptosis in Health and Disease.

[8] Ran Q., Mozolewska P., Tang D. (2019). Gpx4 and Ferroptosis. Ferroptosis in Health and Disease.

[9] Harris I.S., DeNicola G.M. (2020). The Complex Interplay between Antioxidants and ROS in Cancer. Trends Cell Biol..

[10] Tang D. (2019). Ferroptosis in Health and Disease.

[11] Ursini F., Maiorino M. (2020). Lipid peroxidation and ferroptosis: The role of GSH and GPx4. Free. Radic. Biol. Med..

[12] Conrad M., Sato H. (2011). The oxidative stress-inducible cystine/glutamate antiporter, system x c − : Cystine supplier and beyond. Amino Acids.

[13] Newstead S. (2015). Molecular insights into proton coupled peptide transport in the PTR family of oligopeptide transporters. Biochim. Biophys. Acta BBA Gen. Subj..

[14] Bannai S., Kitamura E. (1980). Transport Interactionof t-Cystine and L-Glutamatein Human Diploid Fibroblasts in Culture. Biol. Chem..

[15] Lewerenz J., Hewett S., Huang Y., Lambros M., Gout P.W., Kalivas P.W., Massie A., Smolders I., Methner A., Pergande M. (2013). The Cystine/Glutamate Antiporter System xc− in Health and Disease: From Molecular Mechanisms to Novel Therapeutic Opportunities. Antioxidants Redox Signal..

[16] Lee J., Kang E.S., Kobayashi S., Homma T., Sato H., Seo H.G., Fujii J. (2017). The viability of primary hepatocytes is maintained under a low cysteine-glutathione redox state with a marked elevation in ophthalmic acid production. Exp. Cell Res..

[17] Hayano M., Yang W.S., Corn C.K., Pagano N.C., Stockwell B.R. (2015). Loss of cysteinyl-tRNA synthetase (CARS) induces the transsulfuration pathway and inhibits ferroptosis induced by cystine deprivation. Cell Death Differ..

[18] Forcina G.C., Dixon S.J. (2019). GPX4 at the Crossroads of Lipid Homeostasis and Ferroptosis. Proteomics.

[19] Yant L., Ran Q., Rao L., Van Remmen H., Shibatani T., Belter J.G., Motta L., Richardson A., Prolla T.A. (2003). The selenoprotein GPX4 is essential for mouse development and protects from radiation and oxidative damage insults. Free. Radic. Biol. Med..

[20] Scheerer P., Borchert A., Kraus N., Wessner H., Gerth C., Höhne W., Kuhn H. (2007). Structural Basis for Catalytic Activity and Enzyme Polymerization of Phospholipid Hydroperoxide Glutathione Peroxidase-4 (GPx4)†,‡,§. Biochemistry.

[21] Gaschler M.M., Andia A.A., Liu H., Csuka J.M., Hurlocker B., Vaiana C.A., Heindel D.W., Zuckerman D.S., Bos P.H., Reznik E. (2018). FINO2 initiates ferroptosis through GPX4 inactivation and iron oxidation. Nat. Chem. Biol..

[22] Tarangelo A., Dixon S.J., Tang D. (2019). Lipid Metabolism and ‘Ferroptosis’. Ferroptosis in Health and Disease.

[23] Lei P., Bai T., Sun Y. (2019). Mechanisms of Ferroptosis and Relations With Regulated Cell Death: A Review. Front. Physiol..

[24] Doll S., Proneth B., Tyurina Y.Y., Panzilius E., Kobayashi S., Ingold I., Irmler M., Beckers J., Aichler M., Walch A. (2017). ACSL4 dictates ferroptosis sensitivity by shaping cellular lipid composition. Nat. Chem. Biol..

[25] Feng H., Stockwell B.R. (2018). Unsolved mysteries: How does lipid peroxidation cause ferroptosis?. PLoS Biol..

[26] Angeli J.P.F., Schneider M., Proneth B., Tyurina Y.Y., Tyurin V., Hammond V.J., Herbach N., Aichler M., Walch A., Eggenhofer E. (2014). Inactivation of the ferroptosis regulator Gpx4 triggers acute renal failure in mice. Nat. Cell Biol..

[27] Gaschler M.M., Hu F., Feng H., Linkermann A., Min W., Stockwell B.R. (2018). Determination of the Subcellular Localization and Mechanism of Action of Ferrostatins in Suppressing Ferroptosis. ACS Chem. Biol..

[28] Wong-Ekkabut J., Xu Z., Triampo W., Tang I.-M., Tieleman D.P., Monticelli L. (2007). Effect of Lipid Peroxidation on the Properties of Lipid Bilayers: A Molecular Dynamics Study. Biophys. J..

[29] Piperno A., Pelucchi S., Mariani R. (2020). Inherited iron overload disorders. Transl. Gastroenterol. Hepatol..

[30] Camaschella C., Nai A., Silvestri L. (2020). Iron metabolism and iron disorders revisited in the hepcidin era. Haematologica.

[31] Daher R., Manceau H., Karim Z. (2017). Iron metabolism and the role of the iron-regulating hormone hepcidin in health and disease. La Presse Médicale.

[32] Kwon M.-Y., Park E., Lee S.-J., Chung S.W. (2015). Heme oxygenase-1 accelerates erastin-induced ferroptotic cell death. Oncotarget.

[33] Sun X., Ou Z., Xie M., Kang R., Fan Y., Niu X., Wang H., Cao L., Tang D. (2015). HSPB1 as a novel regulator of ferroptotic cancer cell death. Oncogene.

[34] Hirayama T., Miki A., Nagasawa H. (2019). Organelle-specific analysis of labile Fe(ii) during ferroptosis by using a cocktail of various colour organelle-targeted fluorescent probes. Metallomics.

[35] Wang S.-J., Li D., Ou Y., Jiang L., Chen Y., Zhao Y., Gu W. (2016). Acetylation Is Crucial for p53-Mediated Ferroptosis and Tumor Suppression. Cell Rep..

[36] Sun X., Ou Z., Chen R., Niu X., Chen D., Kang R., Tang D. (2016). Activation of the p62-Keap1-NRF2 pathway protects against ferroptosis in hepatocellular carcinoma cells. Hepatology.

[37] Suzuki T., Motohashi H., Yamamoto M. (2013). Toward clinical application of the Keap1–Nrf2 pathway. Trends Pharmacol. Sci..

[38] Copple I.M. (2012). The Keap1–Nrf2 Cell Defense Pathway–A Promising Therapeutic Target?. Adv. Pharmacol..

[39] DeNicola G.M., Karreth F.A., Humpton T.J., Gopinathan A., Wei C., Frese K., Mangal D., Yu K.H., Yeo C.J., Calhoun E.S. (2011). Oncogene-induced Nrf2 transcription promotes ROS detoxification and tumorigenesis. Nature.

[40] Liu N., Lin X., Huang C. (2020). Activation of the reverse transsulfuration pathway through NRF2/CBS confers erastin-induced ferroptosis resistance. Br. J. Cancer.

[41] Eleftheriadis T., Pissas G., Liakopoulos V., Liakopoulos V., Stefanidis I. (2019). The H2S–Nrf2–Antioxidant Proteins Axis Protects Renal Tubular Epithelial Cells of the Native Hibernator Syrian Hamster from Reoxygenation-Induced Cell Death. Biology.

[42] Bersuker K., Hendricks J.M., Li Z., Magtanong L., Ford B., Tang P.H., Roberts M.A., Tong B., Maimone T.J., Zoncu R. (2019). The CoQ oxidoreductase FSP1 acts parallel to GPX4 to inhibit ferroptosis. Nature.

[43] Han C., Liu Y., Dai R., Ismail N., Su W., Li B. (2020). Ferroptosis and Its Potential Role in Human Diseases. Front. Pharmacol..

[44] Gao M., Yi J., Zhu J., Minikes A., Monian P., Thompson C.B., Jiang X. (2019). Role of Mitochondria in Ferroptosis. Mol. Cell.

[45] Yuan H., Li X., Zhang X., Kang R., Tang D. (2016). CISD1 inhibits ferroptosis by protection against mitochondrial lipid peroxidation. Biochem. Biophys. Res. Commun..

[46] McCullough K., Bolisetty S. (2020). Ferritins in Kidney Disease. Semin. Nephrol..

[47] Latunde-Dada G.O. (2017). Ferroptosis: Role of lipid peroxidation, iron and ferritinophagy. Biochim. Biophys. Acta BBA Gen. Subj..

[48] Mancias J.D., Wang X., Gygi S.P., Harper J.W., Kimmelman A.C. (2014). Quantitative proteomics identifies NCOA4 as the cargo receptor mediating ferritinophagy. Nature.

[49] Liang C., Zhang X., Yang M., Dong X. (2019). Recent Progress in Ferroptosis Inducers for Cancer Therapy. Adv. Mater..

[50] Louandre C., Ezzoukhry Z., Godin C., Barbare J.-C., Mazière J.-C., Chauffert B., Galmiche A. (2013). Iron-dependent cell death of hepatocellular carcinoma cells exposed to sorafenib. Int. J. Cancer.

[51] Angeli J.P.F., Shah R., Pratt D.A., Conrad M. (2017). Ferroptosis Inhibition: Mechanisms and Opportunities. Trends Pharmacol. Sci..

[52] Zilka O., Shah R., Li B., Angeli J.P.F., Griesser M., Conrad M., Pratt D.A. (2017). On the Mechanism of Cytoprotection by Ferrostatin-1 and Liproxstatin-1 and the Role of Lipid Peroxidation in Ferroptotic Cell Death. ACS Cent. Sci..

[53] Gautheron J., Gores G.J., Rodrigues C.M.P. (2020). Lytic cell death in metabolic liver disease. J. Hepatol..

[54] Macías-Rodríguez R.U., Inzaugarat M.E., Ruiz-Margáin A., Nelson L., Trautwein C., Cubero F.J. (2020). Reclassifying Hepatic Cell Death during Liver Damage: Ferroptosis—A Novel Form of Non-Apoptotic Cell Death?. Int. J. Mol. Sci..

[55] Kullak-Ublick G.A., Andrade R.J., Merz M., End P., Benesic A., Gerbes A.L., Aithal G.P. (2017). Drug-induced liver injury: Recent advances in diagnosis and risk assessment. Gut.

[56] Galmiche A., Tang D. (2019). Ferroptosis in Liver Disease. Ferroptosis in Health and Disease.

[57] Yamada N., Karasawa T., Kimura H., Watanabe S., Komada T., Kamata R., Sampilvanjil A., Ito J., Nakagawa K., Kuwata H. (2020). Ferroptosis driven by radical oxidation of n-6 polyunsaturated fatty acids mediates acetaminophen-induced acute liver failure. Cell Death Dis..

[58] Zou Y., Li H., Graham E.T., Deik A.A., Eaton J.K., Wang W., Sandoval-Gomez G., Clish C., Doench J.G., Schreiber S.L. (2020). Cytochrome P450 oxidoreductase contributes to phospholipid peroxidation in ferroptosis. Nat. Chem. Biol..

[59] Saidi R.F., Kenari S.K.H. (2014). Liver Ischemia/Reperfusion Injury: An Overview. J. Investig. Surg..

[60] Friedman S.L., Neuschwander-Tetri B.A., Rinella M., Sanyal A.J. (2018). Mechanisms of NAFLD development and therapeutic strategies. Nat. Med..

[61] Afonso M.B., Castro R.E., Rodrigues C.M.P. (2019). Processes exacerbating apoptosis in non-alcoholic steatohepatitis. Clin. Sci..

[62] Loguercio C., De Girolamo V., De Sio I., Tuccillo C., Ascione A., Baldi F., Budillon G., Cimino L., Di Carlo A., Di Marino M.P. (2001). Non-alcoholic fatty liver disease in an area of southern Italy: Main clinical, histological, and pathophysiological aspects. J. Hepatol..

[63] Sanyal A.J., Chalasani N., Kowdley K.V., McCullough A., Diehl A.M., Bass N.M., Neuschwander-Tetri B.A., LaVine J.E., Tonascia J., Ünalp A. (2010). Pioglitazone, vitamin E, or placebo for nonalcoholic steatohepatitis. New Engl. J. Med..

[64] Nelson J.E., Wilson L., Brunt E.M., Yeh M.M., Kleiner D.E., Unalp-Arida A., Kowdley K.V., Nonalcoholic Steatohepatitis Clinical Research Network (2011). Relationship between the pattern of hepatic iron deposition and histological severity in nonalcoholic fatty liver disease. Hepatology.

[65] Bonkovsky H.L., Jawaid Q., Tortorelli K., LeClair P., Cobb J., Lambrecht R.W., Banner B.F. (1999). Non-alcoholic steatohepatitis and iron: Increased prevalence of mutations of the HFE gene in non-alcoholic steatohepatitis. J. Hepatol..

[66] Tsurusaki S., Tsuchiya Y., Koumura T., Nakasone M., Sakamoto T., Matsuoka M., Imai H., Kok C.Y.-Y., Okochi H., Nakano H. (2019). Hepatic ferroptosis plays an important role as the trigger for initiating inflammation in nonalcoholic steatohepatitis. Cell Death Dis..

[67] Zhou Z., Ye T.J., Bonavita G., Daniels M., Kainrad N., Jogasuria A., You M. (2019). Adipose-Specific Lipin-1 Overexpression Renders Hepatic Ferroptosis and Exacerbates Alcoholic Steatohepatitis in Mice. Hepatol. Commun..

[68] Wang H., An P., Xie E., Wu Q., Fang X., Gao H., Zhang Z., Li Y., Wang X., Zhang J. (2017). Characterization of ferroptosis in murine models of hemochromatosis. Hepatology.

[69] Duarte T.L., Caldas C., Santos A.G., Silva-Gomes S., Santos-Gonçalves A., Martins M.J., Porto G., Lopes J.M. (2017). Genetic disruption of NRF2 promotes the development of necroinflammation and liver fibrosis in a mouse model of HFE-hereditary hemochromatosis. Redox Biol..

[70] Bray F., Ferlay J., Soerjomataram I., Siegel R.L., Torre L.A., Jemal A. (2018). Global cancer statistics 2018: GLOBOCAN estimates of incidence and mortality worldwide for 36 cancers in 185 countries. CA Cancer J. Clin..

[71] Nie J., Lin B., Zhou M., Wu L., Zheng T. (2018). Role of ferroptosis in hepatocellular carcinoma. J. Cancer Res. Clin. Oncol..

[72] Louandre C., Marcq I., Bouhlal H., Lachaier E., Godin C., Saidak Z., Francois C., Chatelain D., DeBuysscher V., Barbare J.-C. (2015). The retinoblastoma (Rb) protein regulates ferroptosis induced by sorafenib in human hepatocellular carcinoma cells. Cancer Lett..

[73] Ou W., Mulik R.S., Anwar A., McDonald J.G., He X., Corbin I.R. (2017). Low-density lipoprotein docosahexaenoic acid nanoparticles induce ferroptotic cell death in hepatocellular carcinoma. Free. Radic. Biol. Med..

[74] Bai T., Wang S., Zhao Y., Zhu R., Wang W., Sun Y. (2017). Haloperidol, a sigma receptor 1 antagonist, promotes ferroptosis in hepatocellular carcinoma cells. Biochem. Biophys. Res. Commun..

[75] Bebber C.M., Müller F., Clemente L.P., Weber J., Von Karstedt S. (2020). Ferroptosis in Cancer Cell Biology. Cancers.

